# Sodium terbium(III) polyphosphate

**DOI:** 10.1107/S1600536810010093

**Published:** 2010-03-24

**Authors:** Abdelghani Oudahmane, Mohamed Daoud, Boumediene Tanouti, Daniel Avignant, Daniel Zambon

**Affiliations:** a Université Cadi Ayyad, Laboratoire de Physico-Chimie des Matériaux et Environnement, Faculté des Sciences Semlalia, Département de Chimie, BP 2390, 40000, Marrakech, Morocco; b Université Blaise Pascal, Laboratoire des Matériaux Inorganiques, UMR CNRS 6002, 24 Avenue des Landais, 63177 Aubière, France

## Abstract

Single crystals of the title compound, NaTb(PO_3_)_4_, were obtained by solid-state reaction. This compound belongs to type II of long-chain polyphosphates with the general formula *A* ^I^ *B* ^III^(PO_3_)_4_. It is isotypic with the NaNd(PO_3_)_4_ and NaEr(PO_3_)_4_ homologues. The crystal structure is built up of infinite crenelated chains of corner-sharing PO_4_ tetra­hedra with a repeating unit of four tetra­hedra. These chains, extending parallel to [100], are linked by isolated TbO_8_ square anti­prisms, forming a three-dimensional framework. The Na^+^ ions are located in channels running along [010] and are surrounded by six oxygen atoms in a distorted octa­hedral environment within a cut-off distance <2.9 Å.

## Related literature

All Na*Ln*(PO_3_)_4_ polyphosphates reported up to now, where *Ln* is a trivalent rare earth element, belong to type II of long-chain polyphosphates *A* ^I^ *B* ^III^(PO_3_)_4_. For corresponding isotypic crystal structures, see: El Masloumi *et al.* (2005 ▶) and Zhu *et al.* (2006 ▶) for *Ln* = La; Zhu *et al.* (2008 ▶) for Ce and Eu; Horchani-Naifer *et al.* (2009 ▶) for Pr; Koizumi *et al.* (1976 ▶) for Nd; Amami *et al.* (2005 ▶) for Gd; El Masloumi *et al.* (2008 ▶) for Y; Amami *et al.* (2004 ▶) for Ho; Maksimova *et al.* (1988 ▶) for Er. For other isotypic polyphosphates with general composition *A* ^I^ *B* ^III^(PO_3_)_4_, see: Linde *et al.* (1983 ▶) for *AB* = KCe; Naïli *et al.* (2006 ▶) for AgGd; Belam *et al.* (2007 ▶) and Jaoudi *et al.* (2003 ▶); for NaBi. For a review on the crystal chemistry of polyphos­phates, see: Durif (1995 ▶).

## Experimental

### 

#### Crystal data

NaTb(PO_3_)_4_
*M* *_r_* = 497.79
Monoclinic, 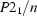

*a* = 7.1712 (1) Å
*b* = 13.0512 (2) Å
*c* = 9.7547 (1) Å
β = 90.604 (1)°
*V* = 912.92 (2) Å^3^
*Z* = 4
Mo *K*α radiation
μ = 8.56 mm^−1^
*T* = 296 K
0.41 × 0.12 × 0.10 mm

#### Data collection

Bruker APEXII CCD diffractometer
Absorption correction: multi-scan (*SADABS*; Bruker, 2008 ▶) *T*_min_ = 0.127, *T*_max_ = 0.478
26640 measured reflections
6971 independent reflections
6445 reflections with *I* > 2σ(*I*)
*R*_int_ = 0.025

#### Refinement

*R*[*F* ^2^ > 2σ(*F* ^2^)] = 0.022
*wR*(*F* ^2^) = 0.055
*S* = 1.10
6971 reflections
163 parameters
Δρ_max_ = 2.12 e Å^−3^
Δρ_min_ = −2.03 e Å^−3^

### 

Data collection: *APEX2* (Bruker, 2008 ▶); cell refinement: *SAINT* (Bruker, 2008 ▶); data reduction: *SAINT*; program(s) used to solve structure: *SHELXS97* (Sheldrick, 2008 ▶); program(s) used to refine structure: *SHELXL97* (Sheldrick, 2008 ▶); molecular graphics: *DIAMOND* (Brandenburg, 1999 ▶); software used to prepare material for publication: *SHELXTL* (Sheldrick, 2008 ▶).

## Supplementary Material

Crystal structure: contains datablocks I, global. DOI: 10.1107/S1600536810010093/wm2313sup1.cif

Structure factors: contains datablocks I. DOI: 10.1107/S1600536810010093/wm2313Isup2.hkl

Additional supplementary materials:  crystallographic information; 3D view; checkCIF report

## Figures and Tables

**Table 1:** Selected bond lengths (Å)

P1—O10	1.4896 (14)
P1—O6	1.4918 (13)
P1—O8^i^	1.5857 (13)
P1—O5	1.5876 (12)
P2—O3^ii^	1.4792 (13)
P2—O4	1.4858 (13)
P2—O8	1.5768 (13)
P2—O2	1.5937 (13)
P3—O11	1.4827 (13)
P3—O1^iii^	1.4868 (13)
P3—O7^iv^	1.5897 (13)
P3—O5	1.5915 (13)
P4—O9^ii^	1.4853 (13)
P4—O12^v^	1.4867 (13)
P4—O2	1.5952 (13)
P4—O7	1.5997 (13)

Symmetry codes: (i) 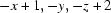; (ii) 
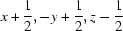
; (iii) 
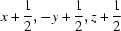
; (iv) 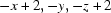; (v) 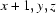.

**Fig. 1.**
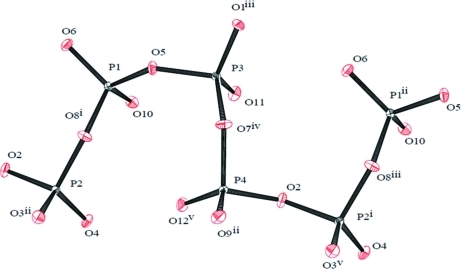
ORTEP view of the repeating unit of the (PO3)∞ chains. Displacement ellipsoids are drawn at the 50% probability level. Symmetry codes: (i) -x+1, -y, -z+2; (ii) x+1/2, -y+1/2, z-1/2; (iii) x+1/2, -y+1/2, z+1/2; (iv) -x+2, -y, -z+2; (v) x+1, y, z.

**Fig. 2.**
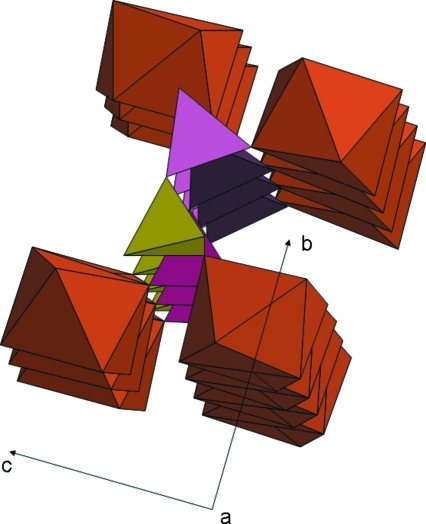
View of four rows of TbO8 polyhedra connected through one (PO3)∞ chain.

**Fig. 3.**
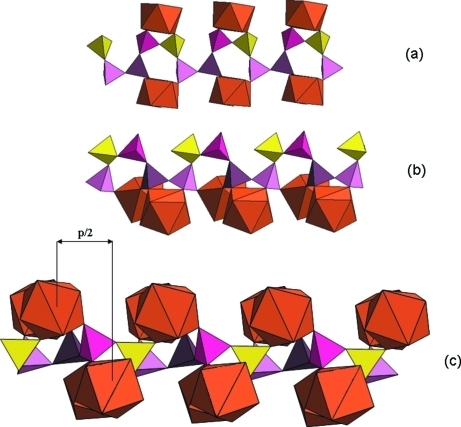
Details of the connections between the (PO3)∞ chains and the TbO8 polyhedra: a) view showing the two kind of bidentate attachments. b) view showing the bidentate attachments and the PO4 groups shared between two TbO8 square antiprisms belonging to two adjacent rows. c) view showing the shift (p/2) of one chain relative to the other.

## supplementary crystallographic information

### Comment

It is now well established that long-chain polyphosphates with general formula *A*^I^*B*^III^(PO_3_)_4_ can be divided in seven structural types (Jaoudi *et al.*, 2003). All long-chain polyphosphates of formula Na*Ln*P_4_O_12_ (*Ln* = rare earth element) reported up to now ( El Masloumi *et al.* 2005; Zhu *et al.* 2006; Zhu *et al.* 2008; Horchani-Naifer *et al.* 2009; Koizumi *et al.* 1976; Amami *et al.* 2005; El Masloumi *et al.* 2008; Amami *et al.* 2004; Maksimova *et al.* 1988) belong to the structural type II which has been first described on basis of the KCe(PO_3_)_4_ structure (Linde *et al.*, 1983). A few other *A*^I^*B*^III^ cationic combinations such as AgGd (Naïli *et al.* 2006) and NaBi (Jaoudi *et al.* 2003; Belam *et al.* 2007) also lead to polyphosphates which belong to the structural type II. The structure of the title compound also fits in this isotypic series. The underlying structure has many times been described as built up of (PO_3_)_∞_ chains running along the [100] direction and further linked by isolated *Ln*O_8_ polyhedra. The resulting three dimensional framework delimits tunnels where the Na^+^ ions are located. Instead of using this description, we will focus on the connectivity between the (PO_3_)_∞_ chains and the TbO_8_ square antiprisms for our account. Each TbO_8_ square antiprism is linked to four (PO_3_)_∞_ chains by corner-sharing involving the non-bridging oxygen atoms of the PO_4_ groups that exhibit the shorter P—O distances within the chain. Their P—O distances range from 1.4792 (13) Å to 1.4918 (13) Å. The chains are crenelated with a repeating unit of four corner-sharing tetrahedra, as displayed in Fig. 1. The repeating unit is built up of PO_4_ tetrahedra corresponding to the four crystallographically independent phosphorus atoms labelled from P1 to P4. If the origin of the chain is taken at the O2 position for instance, then the P2 and P4 tetrahedra are the end-groupings of the repeating unit while P1 and P3 tetrahedra are involved in the internal diphosphate group. Each (PO_3_)_∞_ chain is linked to four rows of isolated TbO_8_ square antiprisms parallel to the direction of the chain (Fig. 2). With the aforementioned origin convention both terminal P(2)O_4_ and P(4)O_4_ tetrahedra are connected in a bidentate fashion on one side of the square face of the archimedean antiprisms of the first row while the internal P(1)O_4_—P(3)O_4_ diphosphate group is also connected in a bidentate fashion on one side of the square face of the antiprisms of the second row (Fig. 3a and 3 b). Therefore the two rows of TbO_8_ polyhedra are translated with a half-period of the (PO_3_)_∞_ chain relative to one another (Fig. 3c). Thus the tetrahedra involved in the internal P_2_O_7_ groups share their non-bridging oxygen atoms with two TbO_8_ polyhedra belonging to each of the first and second rows, respectively. Then the (PO_3_)_∞_ chain is connected to the third and fourth rows in a similar way but the role played by the couples P(1)O_4_—P(3)O_4_ and P(2)O_4_—P(4)O_4_ are inverted, this last becoming the internal diphosphate group.

For a general review on the crystal chemistry of polyphosphates, see: Durif (1995).

### Experimental

Crystals of the title compound were synthesized by reacting Tb_4_O_7_ with (NH_4_)H_2_PO_4_ and Na_2_CO_3_ in a platinum crucible. A mixture of these reagents in the molar ratio 5 : 85 : 10 was used for the synthesis. The mixture has first been heated at 473 K for 12 h, then at 573 K for 12 h and finally at 773 K for 24 h. The muffle furnace was then cooled down first to 723 K at the rate of 2 K^.^h^-1^ and then to room temperature at the rate of 15 K^.^h^-1^. Single crystals were extracted from the batch by washing with hot water.

### Refinement

The highest residual peak in the final difference Fourier map was located 0.61 Å from atom Tb and the deepest hole was located 0.45 Å from atom Tb.

### Crystal data

**Table:**

NaTb(PO_3_)_4_	*F*(000) = 928
*M**_r_* = 497.79	*D*_x_ = 3.622 Mg m^−^^3^
Monoclinic, *P*2_1_/*n*	Mo *K*α radiation, λ = 0.71073 Å
Hall symbol: -P 2yn	Cell parameters from 27177 reflections
*a* = 7.1712 (1) Å	θ = 3.5–43.9°
*b* = 13.0512 (2) Å	µ = 8.56 mm^−^^1^
*c* = 9.7547 (1) Å	*T* = 296 K
β = 90.604 (1)°	Needle, colourless
*V* = 912.92 (2) Å^3^	0.41 × 0.12 × 0.10 mm
*Z* = 4	

### Data collection

**Table:**

Bruker APEXII CCD diffractometer	6971 independent reflections
Radiation source: fine-focus sealed tube	6445 reflections with *I* > 2σ(*I*)
graphite	*R*_int_ = 0.025
Detector resolution: 8.3333 pixels mm^-1^	θ_max_ = 43.8°, θ_min_ = 3.5°
ω and φ scans	*h* = −13→8
Absorption correction: multi-scan (*SADABS*; Bruker, 2008)	*k* = −25→17
*T*_min_ = 0.127, *T*_max_ = 0.478	*l* = −19→18
26640 measured reflections	

### Refinement

**Table:**

Refinement on *F*^2^	0 constraints
Least-squares matrix: full	Primary atom site location: structure-invariant direct methods
*R*[*F*^2^ > 2σ(*F*^2^)] = 0.022	Secondary atom site location: difference Fourier map
*wR*(*F*^2^) = 0.055	*w* = 1/[σ^2^(*F*_o_^2^) + (0.0212*P*)^2^ + 1.888*P*] where *P* = (*F*_o_^2^ + 2*F*_c_^2^)/3
*S* = 1.10	(Δ/σ)_max_ = 0.003
6971 reflections	Δρ_max_ = 2.12 e Å^−^^3^
163 parameters	Δρ_min_ = −2.03 e Å^−^^3^
0 restraints	

### Special details

**Table:**

Geometry. All esds (except the esd in the dihedral angle between two l.s. planes) are estimated using the full covariance matrix. The cell esds are taken into account individually in the estimation of esds in distances, angles and torsion angles; correlations between esds in cell parameters are only used when they are defined by crystal symmetry. An approximate (isotropic) treatment of cell esds is used for estimating esds involving l.s. planes.
Refinement. Refinement of *F*^2^ against ALL reflections. The weighted *R*-factor wR and goodness of fit *S* are based on *F*^2^, conventional *R*-factors *R* are based on *F*, with *F* set to zero for negative *F*^2^. The threshold expression of *F*^2^ > σ(*F*^2^) is used only for calculating *R*-factors(gt) etc. and is not relevant to the choice of reflections for refinement. *R*-factors based on *F*^2^ are statistically about twice as large as those based on *F*, and *R*- factors based on ALL data will be even larger.

### Fractional atomic coordinates and isotropic or equivalent isotropic displacement parameters (Å^2^)

**Table:**

	*x*	*y*	*z*	*U*_iso_*/*U*_eq_	
Na	−0.00068 (15)	0.22179 (9)	1.06373 (11)	0.02105 (19)	
Tb	0.512736 (10)	0.219027 (5)	0.976594 (7)	0.00611 (2)	
P1	0.24948 (6)	0.10137 (3)	1.24460 (4)	0.00585 (6)	
P2	0.87630 (6)	0.11488 (3)	0.76336 (4)	0.00525 (5)	
P3	0.64720 (6)	0.12784 (3)	1.30443 (4)	0.00514 (5)	
P4	1.26813 (6)	0.09081 (3)	0.69983 (4)	0.00584 (6)	
O1	0.22211 (18)	0.28938 (9)	0.89407 (14)	0.01015 (19)	
O2	1.08668 (17)	0.08115 (10)	0.79214 (13)	0.00956 (18)	
O3	0.36683 (19)	0.31058 (10)	1.14928 (13)	0.01003 (18)	
O4	0.79883 (18)	0.14688 (10)	0.89738 (13)	0.00974 (18)	
O5	0.42970 (17)	0.12511 (10)	1.33504 (13)	0.00876 (17)	
O6	0.09345 (18)	0.16584 (10)	1.29625 (14)	0.01081 (19)	
O7	1.28234 (19)	−0.02144 (9)	0.63492 (13)	0.01000 (19)	
O8	0.7833 (2)	0.01335 (10)	0.70784 (14)	0.01089 (19)	
O9	0.73740 (19)	0.33790 (10)	1.08306 (14)	0.01139 (19)	
O10	0.28652 (19)	0.10837 (10)	1.09492 (13)	0.01001 (18)	
O11	0.67772 (19)	0.13293 (10)	1.15449 (13)	0.01040 (19)	
O12	0.42899 (18)	0.11069 (11)	0.79349 (15)	0.0125 (2)	

### Atomic displacement parameters (Å^2^)

**Table:**

	*U*^11^	*U*^22^	*U*^33^	*U*^12^	*U*^13^	*U*^23^
Na	0.0165 (4)	0.0309 (5)	0.0157 (4)	0.0077 (3)	−0.0022 (3)	0.0012 (3)
Tb	0.00563 (3)	0.00740 (3)	0.00529 (3)	0.00035 (2)	0.00018 (2)	−0.00028 (2)
P1	0.00480 (13)	0.00573 (13)	0.00703 (14)	−0.00036 (10)	0.00008 (10)	−0.00060 (11)
P2	0.00561 (13)	0.00468 (12)	0.00548 (13)	0.00011 (10)	0.00126 (10)	0.00071 (10)
P3	0.00532 (13)	0.00475 (12)	0.00534 (13)	−0.00013 (10)	−0.00051 (10)	−0.00029 (10)
P4	0.00542 (13)	0.00510 (13)	0.00700 (14)	0.00056 (10)	0.00050 (10)	0.00007 (11)
O1	0.0087 (4)	0.0083 (4)	0.0134 (5)	0.0018 (3)	−0.0021 (4)	0.0039 (4)
O2	0.0055 (4)	0.0131 (5)	0.0101 (4)	0.0020 (3)	0.0015 (3)	0.0033 (4)
O3	0.0113 (5)	0.0089 (4)	0.0100 (4)	−0.0008 (4)	0.0020 (3)	−0.0047 (4)
O4	0.0090 (4)	0.0131 (5)	0.0071 (4)	0.0025 (4)	0.0029 (3)	−0.0016 (4)
O5	0.0052 (4)	0.0133 (5)	0.0077 (4)	−0.0013 (3)	−0.0003 (3)	−0.0016 (4)
O6	0.0076 (4)	0.0113 (5)	0.0135 (5)	0.0027 (4)	0.0004 (3)	−0.0030 (4)
O7	0.0151 (5)	0.0058 (4)	0.0090 (4)	0.0031 (4)	−0.0026 (4)	−0.0009 (3)
O8	0.0137 (5)	0.0067 (4)	0.0123 (5)	−0.0031 (4)	−0.0005 (4)	−0.0007 (4)
O9	0.0130 (5)	0.0094 (4)	0.0118 (5)	−0.0029 (4)	0.0043 (4)	−0.0046 (4)
O10	0.0121 (5)	0.0105 (4)	0.0074 (4)	−0.0018 (4)	−0.0006 (3)	−0.0001 (3)
O11	0.0106 (5)	0.0140 (5)	0.0066 (4)	0.0017 (4)	0.0009 (3)	0.0027 (4)
O12	0.0070 (4)	0.0156 (5)	0.0149 (5)	−0.0001 (4)	−0.0020 (4)	−0.0064 (4)

### Geometric parameters (Å, °)

**Table:**

Na—O4^i^	2.3680 (17)	P2—O4	1.4858 (13)
Na—O9^i^	2.4223 (17)	P2—O8	1.5768 (13)
Na—O6	2.4700 (17)	P2—O2	1.5937 (13)
Na—O1	2.4750 (18)	P2—Na^vi^	3.3550 (12)
Na—O10	2.5520 (17)	P3—O11	1.4827 (13)
Na—O11^i^	2.7369 (18)	P3—O1^vii^	1.4868 (13)
Na—P1	2.9554 (11)	P3—O7^viii^	1.5897 (13)
Na—O3	2.9897 (17)	P3—O5	1.5915 (13)
Na—P4^ii^	3.2464 (11)	P3—Na^vii^	3.3808 (12)
Na—P2^i^	3.3550 (12)	P4—O9^iv^	1.4853 (13)
Na—P3^iii^	3.3808 (12)	P4—O12^vi^	1.4867 (13)
Tb—O3	2.3232 (12)	P4—O2	1.5952 (13)
Tb—O12	2.3511 (13)	P4—O7	1.5997 (13)
Tb—O11	2.3729 (12)	P4—Na^ix^	3.2464 (12)
Tb—O6^iv^	2.3894 (13)	O1—P3^iii^	1.4868 (12)
Tb—O4	2.3930 (12)	O3—P2^x^	1.4792 (13)
Tb—O1	2.4082 (12)	O4—Na^vi^	2.3680 (17)
Tb—O9	2.4589 (13)	O6—Tb^x^	2.3894 (13)
Tb—O10	2.4676 (13)	O7—P3^viii^	1.5897 (13)
P1—O10	1.4896 (14)	O8—P1^v^	1.5857 (13)
P1—O6	1.4918 (13)	O9—P4^x^	1.4853 (13)
P1—O8^v^	1.5857 (13)	O9—Na^vi^	2.4223 (17)
P1—O5	1.5876 (12)	O11—Na^vi^	2.7369 (18)
P2—O3^iv^	1.4792 (13)	O12—P4^i^	1.4867 (13)
			
O4^i^—Na—O9^i^	81.14 (5)	O1—Tb—O10	78.97 (4)
O4^i^—Na—O6	131.70 (7)	O9—Tb—O10	127.07 (4)
O9^i^—Na—O6	108.55 (6)	O3—Tb—Na^vi^	105.53 (4)
O4^i^—Na—O1	94.62 (6)	O12—Tb—Na^vi^	115.25 (4)
O9^i^—Na—O1	109.61 (6)	O11—Tb—Na^vi^	49.79 (4)
O6—Na—O1	123.18 (6)	O6^iv^—Tb—Na^vi^	85.72 (4)
O4^i^—Na—O10	109.00 (6)	O4—Tb—Na^vi^	40.92 (4)
O9^i^—Na—O10	168.27 (7)	O1—Tb—Na^vi^	155.88 (4)
O6—Na—O10	60.44 (5)	O9—Tb—Na^vi^	42.38 (4)
O1—Na—O10	76.15 (5)	O10—Tb—Na^vi^	122.57 (4)
O4^i^—Na—O11^i^	62.55 (5)	O3—Tb—Na	52.13 (4)
O9^i^—Na—O11^i^	65.37 (5)	O12—Tb—Na	86.28 (4)
O6—Na—O11^i^	78.51 (5)	O11—Tb—Na	108.59 (4)
O1—Na—O11^i^	156.84 (6)	O6^iv^—Tb—Na	113.76 (4)
O10—Na—O11^i^	113.41 (6)	O4—Tb—Na	156.20 (4)
O4^i^—Na—P1	123.36 (5)	O1—Tb—Na	39.78 (4)
O9^i^—Na—P1	138.81 (6)	O9—Tb—Na	122.23 (4)
O6—Na—P1	30.27 (3)	O10—Tb—Na	41.86 (4)
O1—Na—P1	101.34 (5)	Na^vi^—Tb—Na	153.28 (3)
O10—Na—P1	30.27 (3)	O3—Tb—Na^iv^	127.53 (4)
O11^i^—Na—P1	95.15 (4)	O12—Tb—Na^iv^	50.51 (4)
O4^i^—Na—O3	151.45 (6)	O11—Tb—Na^iv^	144.46 (4)
O9^i^—Na—O3	114.70 (6)	O6^iv^—Tb—Na^iv^	33.06 (4)
O6—Na—O3	68.08 (5)	O4—Tb—Na^iv^	76.60 (3)
O1—Na—O3	58.34 (5)	O1—Tb—Na^iv^	65.71 (4)
O10—Na—O3	58.91 (4)	O9—Tb—Na^iv^	107.72 (4)
O11^i^—Na—O3	144.81 (5)	O10—Tb—Na^iv^	124.12 (3)
P1—Na—O3	60.77 (3)	Na^vi^—Tb—Na^iv^	104.11 (3)
O4^i^—Na—P4^ii^	106.40 (5)	Na—Tb—Na^iv^	101.88 (2)
O9^i^—Na—P4^ii^	25.46 (3)	O10—P1—O6	116.01 (8)
O6—Na—P4^ii^	89.03 (4)	O10—P1—O8^v^	111.91 (7)
O1—Na—P4^ii^	109.94 (5)	O6—P1—O8^v^	108.64 (8)
O10—Na—P4^ii^	143.44 (5)	O10—P1—O5	112.35 (7)
O11^i^—Na—P4^ii^	75.60 (4)	O6—P1—O5	108.14 (7)
P1—Na—P4^ii^	117.77 (4)	O8^v^—P1—O5	98.28 (7)
O3—Na—P4^ii^	92.66 (4)	O10—P1—Na	59.71 (6)
O4^i^—Na—P2^i^	22.72 (3)	O6—P1—Na	56.56 (6)
O9^i^—Na—P2^i^	97.54 (5)	O8^v^—P1—Na	125.86 (6)
O6—Na—P2^i^	138.23 (5)	O5—P1—Na	135.51 (6)
O1—Na—P2^i^	74.33 (4)	O3^iv^—P2—O4	117.55 (8)
O10—Na—P2^i^	93.86 (4)	O3^iv^—P2—O8	106.17 (8)
O11^i^—Na—P2^i^	83.77 (4)	O4—P2—O8	112.19 (8)
P1—Na—P2^i^	116.91 (4)	O3^iv^—P2—O2	110.49 (7)
O3—Na—P2^i^	128.89 (4)	O4—P2—O2	106.54 (7)
P4^ii^—Na—P2^i^	122.67 (3)	O8—P2—O2	102.98 (7)
O4^i^—Na—P3^iii^	85.41 (4)	O3^iv^—P2—Na^vi^	113.14 (6)
O9^i^—Na—P3^iii^	86.83 (4)	O8—P2—Na^vi^	139.10 (6)
O6—Na—P3^iii^	140.69 (5)	O2—P2—Na^vi^	73.82 (5)
O1—Na—P3^iii^	23.52 (3)	O11—P3—O1^vii^	119.43 (8)
O10—Na—P3^iii^	99.60 (5)	O11—P3—O7^viii^	110.89 (7)
O11^i^—Na—P3^iii^	139.46 (4)	O1^vii^—P3—O7^viii^	107.72 (7)
P1—Na—P3^iii^	124.20 (4)	O11—P3—O5	109.95 (7)
O3—Na—P3^iii^	72.62 (4)	O1^vii^—P3—O5	104.75 (7)
P4^ii^—Na—P3^iii^	91.96 (3)	O7^viii^—P3—O5	102.65 (7)
P2^i^—Na—P3^iii^	70.71 (2)	O11—P3—Na^vii^	139.71 (6)
O4^i^—Na—Tb^i^	41.45 (3)	O7^viii^—P3—Na^vii^	109.24 (5)
O9^i^—Na—Tb^i^	43.17 (4)	O5—P3—Na^vii^	63.70 (5)
O6—Na—Tb^i^	118.09 (5)	O9^iv^—P4—O12^vi^	118.03 (9)
O1—Na—Tb^i^	118.61 (5)	O9^iv^—P4—O2	111.53 (7)
O10—Na—Tb^i^	143.63 (5)	O12^vi^—P4—O2	107.41 (8)
O11^i^—Na—Tb^i^	41.46 (3)	O9^iv^—P4—O7	106.24 (7)
P1—Na—Tb^i^	136.04 (4)	O12^vi^—P4—O7	110.53 (7)
O3—Na—Tb^i^	157.45 (4)	O2—P4—O7	101.93 (7)
P4^ii^—Na—Tb^i^	66.69 (2)	O12^vi^—P4—Na^ix^	73.95 (7)
P2^i^—Na—Tb^i^	62.75 (2)	O2—P4—Na^ix^	135.52 (6)
P3^iii^—Na—Tb^i^	98.09 (3)	O7—P4—Na^ix^	119.55 (6)
O3—Tb—O12	138.31 (5)	P3^iii^—O1—Tb	141.24 (8)
O3—Tb—O11	86.46 (5)	P3^iii^—O1—Na	114.86 (8)
O12—Tb—O11	113.09 (5)	Tb—O1—Na	101.72 (5)
O3—Tb—O6^iv^	108.96 (5)	P2—O2—P4	130.93 (8)
O12—Tb—O6^iv^	83.15 (5)	P2^x^—O3—Tb	150.04 (8)
O11—Tb—O6^iv^	135.52 (5)	P2^x^—O3—Na	119.88 (7)
O3—Tb—O4	146.18 (5)	Tb—O3—Na	90.04 (4)
O12—Tb—O4	74.40 (5)	P2—O4—Na^vi^	119.28 (8)
O11—Tb—O4	68.11 (4)	P2—O4—Tb	136.43 (8)
O6^iv^—Tb—O4	78.14 (5)	Na^vi^—O4—Tb	97.62 (5)
O3—Tb—O1	69.60 (5)	P1—O5—P3	133.93 (8)
O12—Tb—O1	76.24 (5)	P1—O6—Tb^x^	142.34 (8)
O11—Tb—O1	148.01 (5)	P1—O6—Na	93.17 (7)
O6^iv^—Tb—O1	74.31 (5)	Tb^x^—O6—Na	115.10 (6)
O4—Tb—O1	141.63 (5)	P3^viii^—O7—P4	132.38 (8)
O3—Tb—O9	70.57 (4)	P2—O8—P1^v^	139.15 (9)
O12—Tb—O9	149.47 (5)	P4^x^—O9—Na^vi^	110.04 (8)
O11—Tb—O9	70.76 (5)	P4^x^—O9—Tb	144.13 (8)
O6^iv^—Tb—O9	75.61 (5)	Na^vi^—O9—Tb	94.45 (5)
O4—Tb—O9	79.89 (4)	P1—O10—Tb	128.26 (7)
O1—Tb—O9	117.48 (5)	P1—O10—Na	90.02 (7)
O3—Tb—O10	70.03 (5)	Tb—O10—Na	97.96 (5)
O12—Tb—O10	80.76 (5)	P3—O11—Tb	131.68 (8)
O11—Tb—O10	72.90 (4)	P3—O11—Na^vi^	118.15 (8)
O6^iv^—Tb—O10	151.40 (4)	Tb—O11—Na^vi^	88.75 (5)
O4—Tb—O10	119.40 (5)	P4^i^—O12—Tb	139.65 (8)

Symmetry codes: (i) *x*−1, *y*, *z*; (ii) *x*−3/2, −*y*+1/2, *z*+1/2; (iii) *x*−1/2, −*y*+1/2, *z*−1/2; (iv) *x*+1/2, −*y*+1/2, *z*−1/2; (v) −*x*+1, −*y*, −*z*+2; (vi) *x*+1, *y*, *z*; (vii) *x*+1/2, −*y*+1/2, *z*+1/2; (viii) −*x*+2, −*y*, −*z*+2; (ix) *x*+3/2, −*y*+1/2, *z*−1/2; (x) *x*−1/2, −*y*+1/2, *z*+1/2.
